# Amaranthin-Like Proteins with Aerolysin Domains in Plants

**DOI:** 10.3389/fpls.2017.01368

**Published:** 2017-08-10

**Authors:** Liuyi Dang, Pierre Rougé, Els J. M. Van Damme

**Affiliations:** ^1^Laboratory of Biochemistry and Glycobiology, Department of Molecular Biotechnology, Ghent University Ghent, Belgium; ^2^UMR 152 PHARMA-DEV, Université de Toulouse Toulouse, France

**Keywords:** lectin, amaranthin, aerolysin, toxin, domain architecture, expression analysis

## Abstract

Amaranthin is a homodimeric lectin that was first discovered in the seeds of *Amaranthus caudatus* and serves as a model for the family of amaranthin-like lectins. Though these lectins have been purified and characterized only from plant species belonging to the Amaranthaceae, evidence accumulated in recent years suggests that sequences containing amaranthin domains are widely distributed in plants. In this study, 84 plant genomes have been screened to investigate the distribution of amaranthin domains. A total of 265 sequences with amaranthin domains were retrieved from 34 plant genomes. Within this group of amaranthin homologs, 22 different domain architectures can be distinguished. The most common domain combination consists of two amaranthin domains followed by a domain with sequence similarity to aerolysin. The latter protein belongs to the group of β-pore-forming toxins produced by bacteria such as *Aeromonas* sp. and exerts its toxicity by making transmembrane pores in the target membrane, as such facilitating bacterial invasion. In addition, amaranthin domains also occur in association with five other protein domains, including the fascin domain, the alpha/beta hydrolase domain, the TRAF-like domain, the B box type zinc finger domain and the Bet v1 domain. All 16 amaranthin-like proteins retrieved from the cucumber genome possess a similar domain architecture consisting of two amaranthin domains linked to one aerolysin domain. Based on phylogenetic differences, four sequences were selected for further investigation. Subcellular localization studies revealed that the amaranthin-like proteins from cucumber reside in the cytoplasm and/or the nucleus. Analyses using qPCR showed that the transcript levels for the amaranthin-like sequences are typically low and expression levels vary among tissues during the development of cucumber plants. Furthermore, the expression of amaranthin-like genes is enhanced after different abiotic stresses, suggesting that these amaranthin-like proteins play a role in the stress response. Finally, molecular modeling was performed to unravel the structure of amaranthin-like proteins and their carbohydrate-binding sites. This study provided valuable information on the distribution, phylogenetic relationships, and possible biological roles of amaranthin-like proteins in plants.

## Introduction

Lectins are proteins with one or more non-catalytic domains that can recognize and bind reversibly with specific mono- or oligosaccharides (Peumans and Van Damme, 1995). They are present in all kingdoms of life and play vital roles in many biological processes (Lichtenstein and Rabinovich, 2013; Lannoo and Van Damme, 2014; Andre et al., 2015). Although they are ubiquitous in nature, the majority of lectins have been characterized from plants. Since the discovery of the first plant lectin more than 100 years ago, several hundreds of plant lectins have been studied in great detail (Van Damme, 2014). With the discovery of the many lectins from diverse origins, plant lectins have been classified into 12 families based on the sequence of their carbohydrate-recognition domains (Van Damme et al., 2008; Lannoo and Van Damme, 2014). So far, the amaranthin family is considered as one of the smallest plant lectin families.

The first lectin from the amaranthin family was purified from the seeds of *Amaranthus caudatus* and represents a 66 kDa homodimeric protein further referred to as amaranthin. The three-dimensional structure of the *A. caudatus* agglutinin was resolved and revealed two carbohydrate-binding sites located at the interface of the two subunits (Transue et al., 1997). In recent years, sequences homologous to amaranthin have also been identified outside the family Amaranthaceae, suggesting that the occurrence of amaranthin-like proteins is much wider and more amaranthin-like proteins remain to be discovered in the plant kingdom. Fortunately, publicly available genome databases enabled us to extend the knowledge about the amaranthin-like sequences in a wide range of species and understand the distribution of amaranthin-like proteins in different plant families.

Our previous study revealed 16 sequences containing amaranthin domains in the cucumber (*Cucumis sativus* L.) genome (Dang and Van Damme, 2016). All these amaranthin homologs from cucumber possess an AAT domain arrangement, consisting of two amaranthin domains (the AA domain) linked to one toxin domain (the T domain), with sequence similarity to aerolysin, the pore-forming toxin (PFT) produced by *Aeromonas* sp. An important feature of these pore-forming proteins is that they are synthesized as soluble proteins that subsequently oligomerize and convert to transmembrane pores in the target membrane (Iacovache et al., 2010). The most extensively studied pore-forming proteins are bacterial PFTs, which have been classified as α- and β-PFTs based on the secondary structure of the toxin domain building the pore, either α-helices or β-hairpins organized in a β-barrel (Geny and Popoff, 2006). Aerolysin, produced by *Aeromonas* sp., is the founding member of the β-PFTs (Szczesny et al., 2011). It is synthesized as a 52 kDa, biologically inactive precursor called proaerolysin, which is only activated after removal of the C-terminal peptide (Parker et al., 1994). So far, aerolysin domains have been reported in proteins from all kingdoms of life. Cluster mapping and phylogenetic analyses have shown that horizontal gene transfer might have played a significant role in the evolution of aerolysins (Moran et al., 2012). In eukaryotes, the aerolysin-like proteins are composed of an N-terminal lectin domain followed by the aerolysin pore-forming domain (Manzano et al., 2017). Till now, proteins with AAT domain architectures have been reported in wheat, flax and *Rumex acetosa* (Puthoff et al., 2005; Faruque et al., 2015; Manzano et al., 2017). The question remains how widely sequences with AAT domain architectures are distributed in the plant kingdom.

To study the distribution of amaranthin domains in plants and their combinations with aerolysin domains, an extensive screening was conducted across a wide range of plant genomes to identify sequences containing amaranthin domains. Four amaranthin-related sequences with AAT domain architectures from cucumber were selected for more in-depth analysis. Microscopical analyses of enhanced green fluorescent protein (EGFP) fusion proteins allowed to study the subcellular localization for these AAT-like proteins in the cell. The transcript levels for these AAT-like genes were evaluated in several tissues during cucumber development under normal growth conditions as well as in the presence of several abiotic stresses or hormone treatments. In addition, the phylogenetic relationships between these AAT-like genes and other aerolysin-like genes were analyzed. Finally, molecular modeling was performed to give insight into the structure of amaranthin-like proteins and their possible carbohydrate-binding properties.

## Materials and Methods

### Identification of Amaranthin Domains in Different Plant Genomes

The protein sequence for amaranthin (GenBank: AAL05954) was used to perform BLASTp searches against different plant genome databases (Supplementary File 1) with default settings to identify sequences containing amaranthin domains (pfam: Agglutinin/PF07468). BLASTp searches were repeated with the top hits to obtain all the possible candidate sequences. The sequences retrieved were then screened with InterProScan 5 for the presence of amaranthin domains and other protein domains attached (Mulder and Apweiler, 2007). Different domain architectures were summarized according to the results of InterProScan 5.

The presence of signal peptides and transmembrane regions was analyzed with the SignalP 4.1 server and TMHMM Server v.2.0, respectively (Krogh et al., 2001; Petersen et al., 2011). The taxonomic tree of plant species was generated using PhyloT^1^ based on the NCBI taxonomy and visualized through Interactive Tree of Life (iTOL^2^) (Letunic and Bork, 2016).

### Phylogenetic Analysis of AA Domains and Aerolysin Domains from Amaranthin-Like Proteins

The tandem arrayed amaranthin (AA) domain sequences from all identified amaranthin-like proteins in different plant genomes were used for the phylogenetic analysis of AA domains. Similarly, sequences encoding the toxin (T) domains were used for the phylogenetic analysis of aerolysin domains (Pfam: PF01117). Aerolysin domains from different cucumber AAT proteins as well as from proteins with similar domain architectures were included in the analysis: Hfr-2 (GenBank: AAW48295) and Dln1 (PDB: 4ZNR_A) from the Pfam database; aerolysin (GI: 113485), LSLa (GI:241161788), LSLb (GI:32261218), LSLc (GI:32261220) from Moran et al. (2012).

Multiple sequence alignment was conducted with MUSCLE using the default settings (Edgar, 2004). After trimming with trimAL, the alignment was used to build a phylogenetic tree with RAxML v8 available from CIPRES Science Gateway according to the maximum likelihood method (Stamatakis et al., 2008; Capella-Gutierrez et al., 2009). The bootstrap iterations were decided automatically by RAxML. The phylogenetic tree was displayed and edited with MEGA6 (Hall, 2013).

Reconciliation of the phylogenetic tree with the species tree was performed in Notung 2.9 (Stolzer et al., 2012). The species tree, containing all species from which amaranthin domains were analyzed, was constructed in NCBI taxonomy^3^.

### Construction of the EGFP-Fusion Vectors for Expression Analysis in Tobacco Cells

Plasmids for expression of the amaranthin-like sequences N- or C-terminally fused to EGFP under the control of the CaMV 35S promoter were constructed using the Gateway Cloning technology (Invitrogen). Coding sequences were amplified as attB PCR products using cDNA obtained from RNA extracted from 9-day-old plant leaves from cucumber (*C. sativus* L. cv. Vert Petit de Paris) except for AAT14, which was amplified from cDNA obtained from 8-day-old fruits. To obtain a complete attB PCR product, nested PCR was performed. The primers used for the PCR are shown in Supplementary Table 1. Amplifications were done with or without stop codon in case of C-terminal or N-terminal fusion to EGFP, respectively. The second PCR was performed using 1:10 diluted product from first PCR as the template. The PCR program was as follows: 5 min at 94°C, 30 cycles (30 s at 94°C, 30 s at 50°C, 1.5 min at 72°C), 5 min at 72°C. Subsequently, the BP reaction was performed using the pDONR221 vector (Invitrogen). After sequencing of the entry clones, the LR reaction was done using pK7WGF2 and pK7FWG2 as destination vector to fuse the amaranthin-like sequences C-terminally or N-terminally to EGFP, respectively (Karimi et al., 2002).

### Transient Transformation of EGFP Fusion Proteins

The EGFP fusion constructs were transferred into *Agrobacterium* strain C58 pMP90 or GV3101 by triparental mating. Six-week-old tobacco leaves (*Nicotiana benthamiana*) were infiltrated by suspensions of *Agrobacterium tumefaciens* with different OD_600_ (0.05–0.2) containing EGFP fusion constructs. The tobacco leaves were checked 2 days after the infiltration by confocal microscopy. The images of tobacco leaves expressing the fluorescent proteins were acquired using a Nikon A1R confocal system, mounted on a Nikon Ti microscope body. Different fluorescent images were acquired along the *z*-axis to create a picture of the complete cell. The acquired images were analyzed by Fiji software^4^.

### Expression Analysis of Cucumber AAT Genes in Different Tissues During Plant Development

Cucumber (*C. sativus* L. cv. Vert Petit de Paris) seeds were germinated on moist filter paper in a Petri dish for 2 days at 28°C in the dark. Germinated seedlings were transferred to pots containing commercial soil, and grown in a plant growth room at 28°C with a 16 h/8 h light/dark photoperiod. Samples of cotyledons, leaves, stems, roots, flower buds, and fruits (collected at 0, 4, 8, 12, and 16 days after pollination) were collected from plants at different stages of plant development. All experiments were performed between April 2013 and September 2014.

Total RNA was isolated from different samples using TRIzol reagent (Sigma–Aldrich) and treated with DNase I (Thermo Fisher Scientific) to remove any traces of genomic DNA according to the manufacturer’s instructions. The first strand cDNA synthesis was performed using M-MLV reverse transcriptase (Thermo Fisher Scientific). RNA concentrations were measured with Nanodrop 2000 spectrophotometer (Thermo Fisher Scientific). The quality of the cDNA was checked by standard RT-PCR using primers of reference genes.

Real-time quantitative PCR analyses were performed using Rotor-Gene 3000 using Rotor Discs (Corbett Life Science, Qiagen). The qPCR reactions were carried out in a total volume of 20 μl containing 10 μl of SYBR Green PCR Sensi-Mix (Bioline), 1 μl of each primer (10 μmol/μl), 1 μl of cDNA template (20 ng/μl), and 7 μl of sterile distilled water. The conditions for qPCR reaction were 96°C for 10 min followed by 45 cycles at 96°C for 25 s, 58°C for 25 s and 72°C for 20 s. Gene-specific primers for qPCR were designed using Primer 3^5^ for amplification of a 100–200 bp fragment. The primers for target and reference genes (clathrin adapter complex subunit and Protein phosphatase 2A) for qPCR analysis are available in Supplementary Table 2 (Migocka and Papierniak, 2011). The results of qPCR were analyzed using Relative Expression Software Tool-384 version 2 (REST-384), which also determined the statistical significance of the results (Pfaffl et al., 2002). All experiments were performed with two independent biological replicates, each containing three technical replicates.

### Responsiveness of Cucumber AAT Genes toward Abiotic Stress/Hormone Treatments

Cucumber (*C. sativus* L. cv. Vert Petit de Paris) seeds were germinated on moist filter paper in Petri dishes at 28°C in the dark. Afterwards, germinated seedlings were transferred to falcon tubes containing half-strength Hoagland solution and grown at 28°C with a 16/8 h light/dark photoperiod. Cucumber seedlings with two fully expanded leaves were used for abiotic stress treatments. For the low temperature treatment, the plants were placed at 4°C in dark. For salt, drought and abscisic acid (ABA) treatments, plants were kept in half-strength Hoagland solution containing 200 mM NaCl, 100 mM mannitol or 100 μM ABA, respectively. All stress treatments were applied for 1, 3, 6, 12, and 24 h. After treatment, the second expanded leaves from cucumber plants were harvested and immediately frozen in liquid nitrogen.

Total RNA extractions and DNase I treatment were performed as described above. The first-strand cDNA synthesis was performed using Maxima Reverse Transcriptase (Thermo Fisher Scientific) according to manufacturer’s instructions. RNA concentrations were measured with Nanodrop 2000 spectrophotometer. The quality of the cDNA was checked by standard RT-PCR using primers of reference genes.

Real-time quantitative PCR analyses were performed using CFX Connect Real-Time PCR Detection Systems (Bio-Rad). The qPCR reactions were carried out in a total volume of 20 μl containing 10 μl of SYBR Green Supermix (Bio-Rad), 1 μl of each primer (10 μmol), 1 μl of cDNA template (20 ng/μl), and 7 μl of sterile distilled water. The conditions for qPCR were 96°C for 10 min followed by 45 cycles at 96°C for 25 s, 58°C for 25 s and 72°C for 20 s. The clathrin adapter complex subunit gene and protein phosphatase 2A gene were used as reference genes for qPCR analysis. The stability of these reference genes was analyzed by qBase PLUS software (Hellemans et al., 2007). REST-384 software was used to analyze the qPCR results and determine the statistical significance (Pfaffl et al., 2002). All experiments were performed with two independent biological replicates, each containing two technical replicates.

### Molecular Modeling of AAT4

The cucumber lectin AAT4 was modeled using the X-ray coordinates of amaranthin from *A. caudatus* (PDB code 1JLY; Transue et al., 1997), the aerolysin-like protein Dln1 from zebrafish (*Danio rerio*, PDB code 5DI0; Jia et al., 2016), the hemolytic lectin from the mushroom *Laetiporus sulphureus* (PDB code 1W3F; Mancheno et al., 2005), the parasporin-2 toxin from *Bacillus thuringiensis* (PDB code 2ZTB) (Akiba et al., 2009), and the Cry23Aa1/Cry37Aa1 toxin complex of *B. thuringiensis* (4RHZ) as templates. Homology modeling of AAT4 was performed with the YASARA Structure program (Krieger et al., 2002), running on a 2.53 GHz Intel duo core Macintosh computer. Nine residues (Leu27, Phe28, Arg141, Asp220, Asn248, Ser326, Lys328, Glu329, Asp369) out of 518 amino acids, of the hybrid model built for AAT4, occurred in the non-allowed regions in the Ramachandran plot. Using ANOLEA to evaluate the hybrid model, 10 residues (over 466) of the AAT4 model exhibited an energy value over the threshold. All of these residues occur in loop regions connecting the β-sheets in the model. The calculated QMEAN6 score of the model gave a value of 0.543.

Docking experiments were performed with the YASARA structure program. Some docking experiments were performed at the SwissDock web server^6^ (Grosdidier et al., 2011a,b), as a control for our docking experiments. Molecular cartoons were drawn with YASARA and Chimera (Pettersen et al., 2004).

## Results

### Occurrence of Proteins Containing One or More Amaranthin Domains in Plant Genomes

**FIGURE 1:**
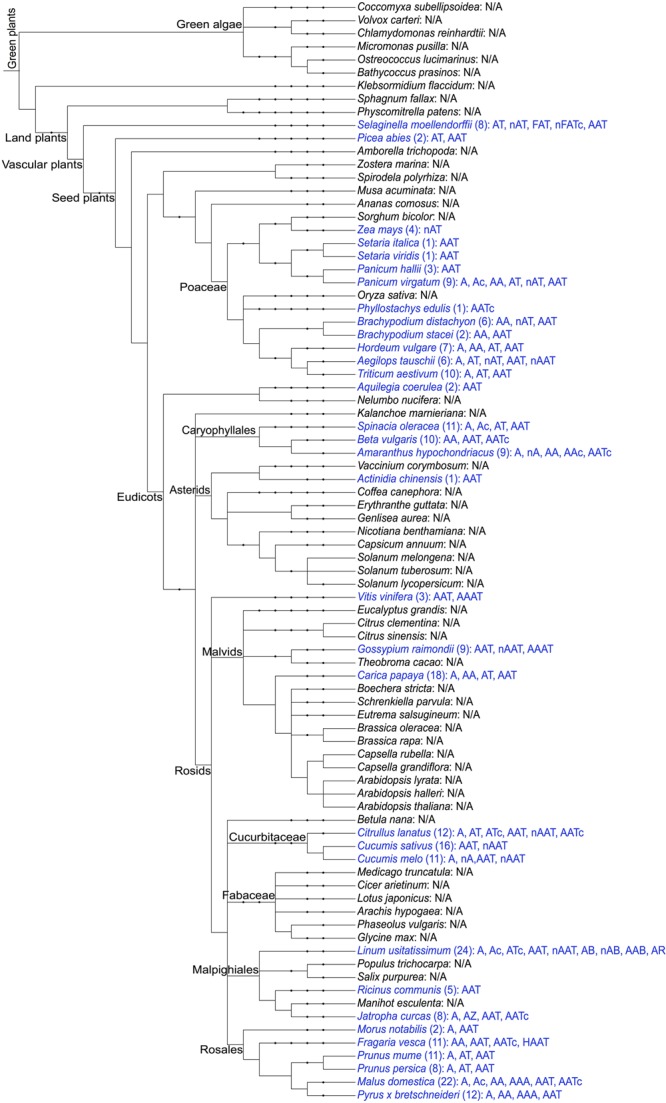
Schematic overview of the distribution of amaranthin domains in the sequenced plant genomes. The taxonomic tree of plant species was generated using PhyloT (http://phylot.biobyte.de/) based on the NCBI taxonomy and visualized through Interactive Tree of Life (iTOL: http://itol.embl.de/). The plant species with amaranthin domains are shown in blue and the number of sequences and their domain architectures are indicated after the names. N/A refers to not applicable, indicating that no amaranthin domain was retrieved from this species. Abbreviations in the domain architectures—n, N-terminal domain (>50 aa); c, C-terminal domain (>50 aa); A, amaranthin domain; T, aerolysin domain; F, fascin domain; B, Bet v1 domain; R, TRAF-like domain; H, alpha/beta hydrolase domain; Z, B-box type zinc finger domain.

The presence of amaranthin domains was investigated in 84 (nearly) completed plant genomes and is summarized in **Figure 1**. In total, 264 sequences with amaranthin domains were identified from 33 plant genomes (not including amaranthin from *A. caudatus*), including 11 monocot plants and 20 dicot plants, as well as two other species (*Selaginella moellendorffii* from Lycopodiophyta, *Picea abies* from Pinophyta). Details of the sequences are available from Supplementary Table 3. The results show that the amaranthin domains are widely distributed among vascular plants, including monocots, dicots, and non-flowering plants. Even in the ancient vascular plant *S. moellendorffii*, multiple sequences with different domain architectures were identified. Obviously, amaranthin domains are not ubiquitous in the plant kingdom. They are absent from more than half of the plant genomes studied (51 out of 84 plant species) and were not found in the six species belonging to green algae.

### Domain Architectures for Sequences with Amaranthin Domains

**FIGURE 2:**
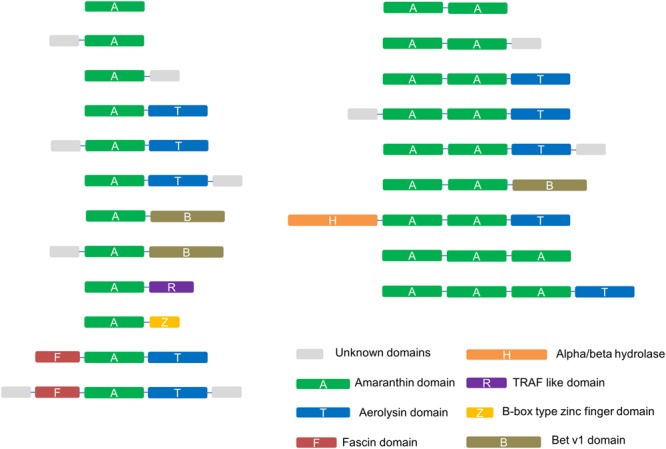
Schematic representation of the identified amaranthin domain architectures in 34 plant genomes. Unknown domains are sequences longer than 50 amino acids at the N-terminus or C-terminus that show no sequence homology to any protein domain with known function.

Sequence analyses revealed different domain arrangements among the sequences with amaranthin domains. Databases such as Pfam and Superfamily were also checked to obtain more comprehensive information about the protein domains. Based on all information retrieved, 21 types of domain architectures can be distinguished in all identified sequences (**Figure 2**). The most prevalent domain combination consists of two amaranthin domains in combination with one aerolysin domain (AAT) and was identified in 113 out of 265 identified sequences. Other protein domains identified in combination with an amaranthin domain include the fascin domain, the Bet v1 domain, the TRAF-like domain, an alpha/beta hydrolase domain and the B box type zinc finger domain.

Sequence analyses suggested that almost all amaranthin proteins are synthesized without a signal peptide and do not contain any transmembrane region. Only two amaranthin-like proteins from *Malus domestica* (AATc domain architecture) and *Fragaria vesca* (AAT domain architecture) were predicted to possess a transmembrane region, located at the C-terminal end of the unknown protein domain.

Not considering the unknown domains, the amaranthin family can generally be divided into six types of domain architectures. (1) Single-domain amaranthin-like proteins (A type); (2) proteins with a single amaranthin domain linked to another domain, e.g., aerolysin domain (A+ type); (3) double amaranthin domain proteins (AA type); (4) proteins with two amaranthin domains in combination with another domain (AA+ type); (5) proteins with triple amaranthin domains (AAA type); (6) proteins containing three amaranthin domains and one aerolysin domain (AAA+ type). Due the diversity in protein structures, different types of amaranthin-like polypeptides differ greatly in size, with calculated molecular masses ranging from 19.5 kDa (A type) to 69.7 kDa (AAA+ type).

**FIGURE 3:**
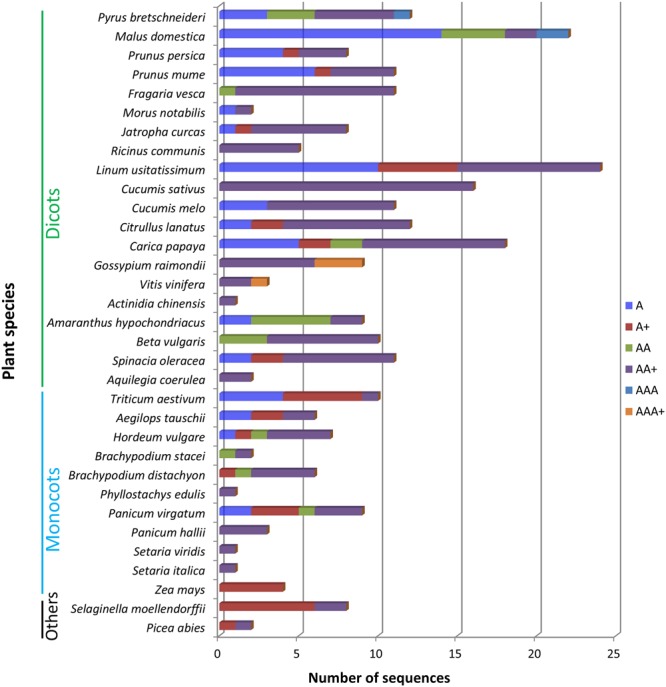
Different types of domain architectures in studied plant genomes. A, single amaranthin domain proteins; A+, proteins with single amaranthin domain linked with another protein domain; AA, double amaranthin domain proteins; AA+, proteins with double amaranthin domains in combination with another protein domain; AAA, proteins with triple amaranthin domains; AAA+, proteins containing three amaranthin domains and one aerolysin domain.

The differences in the abundancy of domain architectures between different plant species are remarkable. According to **Figure 3**, the top 4 plant genomes with the largest number of amaranthin-like sequences are flax (*Linum usitatissimum*), apple (*M. domestica*), papaya (*Carica papaya*), and cucumber (*C. sativus*). Some amaranthin domains from flax are linked to a Bet v1 domain or a TRAF-like domain, a combination that was not found in any other studied plant genome. The apple genome harbors the highest number of sequences with a single amaranthin domain and the cucumber genome has most sequences with an AAT domain organization.

### Phylogenetic Analysis of Amaranthin-Like Proteins in Plants

**FIGURE 4:**
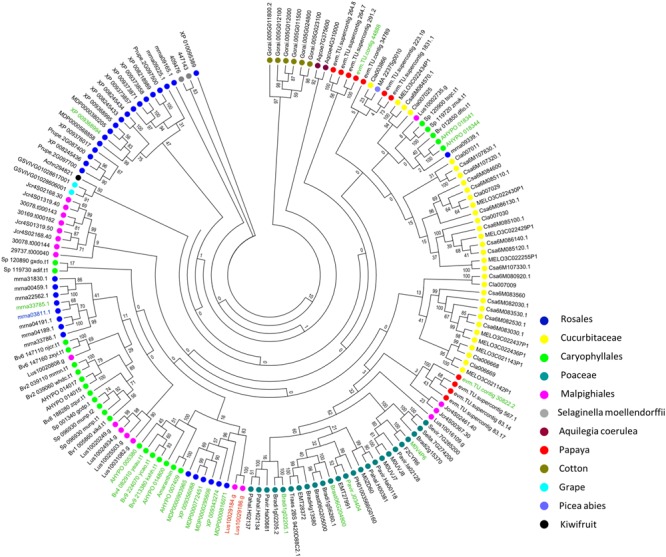
Phylogenetic analysis of sequences encoding two amaranthin domains from all identified amaranthin-like genes. Color spots before gene accession numbers represent the different plant species. The different colors of the accession numbers reflect the domain architecture of the sequences: AAT/nAAT/AATc are shown in black, nAA/AAc shown in green, HAAT shown in blue, and AAB shown in red.

Because of the low sequence similarity between single amaranthin domains, a phylogenetic analysis was conducted using 159 sequences containing two tandem arrayed amaranthin domains to investigate the evolutionary relationships between amaranthin-like proteins (**Figure 4**). In general, amaranthin homologs clustered according to the species they were retrieved from. This dendrogram basically reflects the phylogeny of the plant species (as shown in **Figure 1**). Different conclusions can be drawn from the phylogenetic analysis. First, sequences from species belonging to the same plant family are often clustered together, such as, e.g., Cucurbitaceae (*Citrullus lanatus, C. sativus*, and *Cucumis melo*) and Poaceae (*Setaria italica, Setaria viridis, Panicum hallii, Panicum virgatum, Phyllostachys edulis, Brachypodium distachyon, Brachypodium stacei, Hordeum vulgare, Aegilops tauschii*, and *Triticum aestivum*). Second, sequences with the AA domain architecture, including the amaranthin from *A. caudatus*, were mainly found in Caryophyllales, Rosales, and Poaceae and are mostly separated from AA sequences which are part of the AAT domain architecture. Third, several genes from distantly related species show higher sequence similarities than genes from more closely related species. For example, two genes from flax (Lus10029184.g and Lus10029186.g with AAB domain) are clustered with genes of AA domains from Rosales rather than other genes from flax. This indicated that flax genes with AAB domains might be evolved from genes with AA domains by obtaining an additional Bet v1 domain.

### Amaranthin-Like Proteins in Cucumber

**FIGURE 5:**
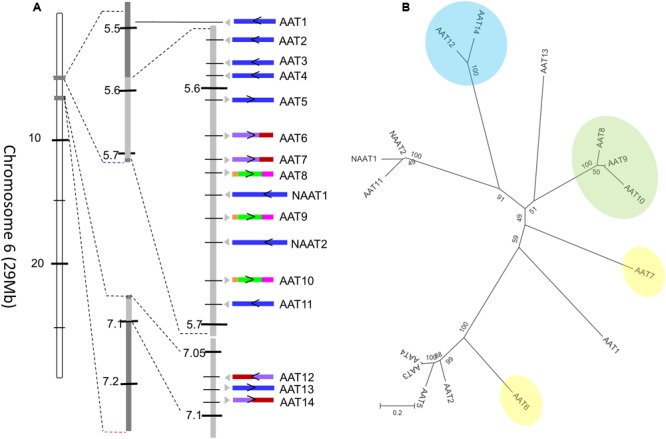
Amaranthin-like proteins in *C. sativus*. **(A)** Distribution of AAT sequences on chromosome 6 of cucumber. The arrow indicates the direction of the gene on the chromosome. The intron/exon composition is shown with colors. Sequences without intron are colored in blue; sequences with one intron are in purple (the first exon) and red (the second exon); sequences with two introns are in orange (the first exon), green (the second exon), and pink (the third exon). **(B)** Phylogenetic tree of all amaranthin-like genes in cucumber. Sequences with similar intron/exon composition are marked with same color. Numbers next to nodes indicate bootstrap values.

The most prevalent domain architecture among all the plant species studied harbors two amaranthin domains linked to one aerolysin toxin domain (AAT), and was most abundant in the cucumber genome. In total, 16 sequences with an AAT domain architecture have been identified. Two of these sequences possess an N-terminal domain with unknown function. In our study, AAT genes of cucumber were numbered sequentially according to their location on the chromosome and domain architecture. Consequently, the AAT proteins from cucumber are referred to as AAT1 to AAT14, and NAAT1 and NAAT2 refer to the sequences containing an additional N-terminal domain. All 16 genes are localized in the first 7.1 Mb of chromosome 6 (total size: 29 Mb; **Figure 5A**).

A phylogenetic analysis with all AAT sequences from cucumber yielded four major clades (**Figure 5B**). Interestingly, these clades are in agreement with the intron/exon composition of the sequences. Seven out of 16 genes contain one or two intron sequences. The sequences AAT8, AAT9, and AAT10 contain two introns located in the first amaranthin domain and the aerolysin domain, respectively (**Figure 5B**, clade indicated in green). The sequences AAT12 and AAT14 harbor a single intron in the middle of the second amaranthin domain (**Figure 5B**, clade indicated in blue) while AAT6 and AAT7 have one intron within the toxin domain (**Figure 5B**, sequences indicated in yellow). However, the position of the intron sequences is not conserved. All other AAT genes have no intron sequence. Our previous results have shown that tandem duplication is the main driver for the expansion of amaranthin-like genes in cucumber (Dang and Van Damme, 2016). Reconciliating the amaranthin domain tree with the species tree gave insights into the duplication events that occurred (Supplementary Figure 1). The results indicated that multiple duplication events took place before species diversification between *C. sativus, C. melo*, and *C. lanatus*. After the diversification of species, the number of AAT genes in cucumber still increased as a result of additional duplication events.

In this study, four genes (AAT4, AAT9, AAT14, and NAAT1) representing one sequence from each major clade of the tree were selected for further analysis.

### Subcellular Localization of AAT Proteins from Cucumber

To investigate the subcellular localization of the amaranthin-like proteins from cucumber, EGFP fusion constructs were made and transformed into *N. benthamiana* leaf epidermal cells. Fluorescence throughout the cells was analyzed using confocal microscopy.

**FIGURE 6:**
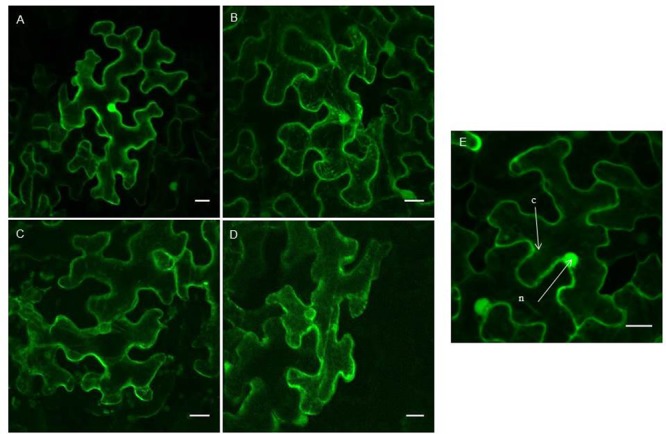
Confocal images of EGFP fusion proteins for different AAT sequences from cucumber. **(A–D)** Show transiently transformed tobacco leaves with expression of EGFP-AAT4 **(A)**; AAT4-EGFP **(B)**; EGFP-NAAT1 **(C)**; NAAT1-EGFP **(D)**. **(E)** Shows a control experiment with free EGFP fluorescence in the cytoplasm and nucleus. Scale bars represent 20 nm. Cell compartments: n, nucleus; c, cytoplasm.

Transient expression of the AAT4 constructs in tobacco leaves yielded strong fluorescence in the nucleus and the cytoplasm (**Figures 6A,B**). Similar results were obtained for N- and C-terminal fusion constructs with EGFP. The expression of AAT4 in the nucleus and the cytoplasm agrees with the prediction that the AAT4 sequence does not contain a signal peptide or a transmembrane region. The two EGFP fusion constructs for AAT9 yielded similar results as for AAT4, with fluorescence in the nucleus and the cytoplasm (Supplementary Figure 2A). In contrast, transient expression of both NAAT1 constructs showed fluorescence in the cytoplasm and around the nucleus (**Figures 6C,D**). Z-stack images clearly showed that the NAAT1 is not expressed in the nucleus. DAPI counterstaining confirmed that the nucleus is free of fluorescence (Supplementary Figure 3). A construct for free EGFP was used as a control and showed bright fluorescence in the nucleus (**Figure 6E**).

Since no fluorescence was observed for the two EGFP constructs of AAT14 when transiently expressed in tobacco leaf epidermal cells, stable transformation of the constructs in *Nicotiana tabacum* cv BY-2 cells was performed. Expression of AAT14-EGFP yielded fluorescence in the cytoplasm but not in the nucleus (Supplementary Figure 2B), suggesting a subcellular localization for AAT14 similar to that of NAAT1.

### Expression Analysis of AAT Genes in Different Cucumber Tissues throughout Cucumber Development

Transcript levels for the AAT genes under study were determined for various developmental stages and tissues of cucumber plants, including cotyledons, leaves, stems, roots, flowers, and fruits. Samples were collected at different developmental stages of cucumber: germinating seeds (4 days), plants with first true leaf (9 days), plants that start flowering (51 days), and fruits collected at different stages of maturation. For all samples, transcripts levels for the AAT genes were quantified and normalized against reference genes. Relative expression levels of AAT genes from vegetative organs (leaves, stems, roots) were compared to the expression level in cotyledons while relative expression levels of AAT genes from reproductive organs (flowers and fruits) were compared to the expression level in flowers.

**FIGURE 7:**
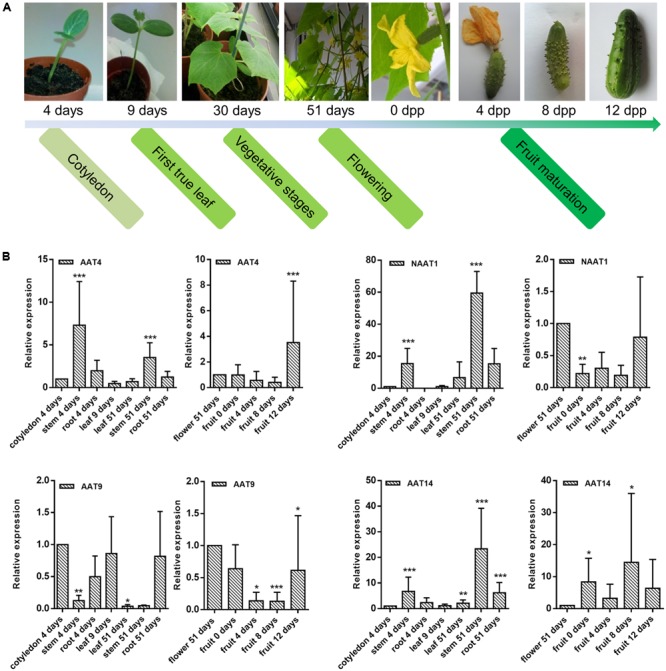
Transcriptional profiling of AAT genes in different tissues during cucumber development. **(A)** Overview of development stages of cucumber plants for sample collections. **(B)** Relative expression levels of AAT genes from vegetative organs (leaves, stems, roots) are compared to the expression level in cotyledons while relative expression levels of AAT genes from reproductive organs (flowers and fruits) are compared to the expression level in flowers. Expression levels compared between cotyledon and flower: AAT4 (cotyledon):AAT4 (flower) = 1:0.8; NAAT1 (cotyledon):NAAT1 (flower) = 1:59.6; AAT9 (cotyledon):AAT9 (flower) = 1:0.14; AAT14 (cotyledon):AAT14 (flower) = 1:0.7. Expression levels compared between four AAT genes in cotyledon: AAT4:NAAT1:AAT9:AAT14 are approximately 1:0.01:1.49:0.04. Bars represent means and standard errors from two biological replicates, each replicate containing a pool of three plants. Asterisks indicated statistically significant differences compared with the control tissue (^∗^*p* ≤ 0.05, ^∗∗^*p* ≤ 0.01, ^∗∗∗^*p* ≤ 0.001; REST analysis).

Different expression profiles were observed for the different AAT genes under study (**Figure 7** and Supplementary Figure 4). Transcripts encoding AAT4 were present continuously in all samples at different stages of cucumber development, with the highest expression observed in stems (at day 4 and 51 of development) and fruits (12 days after pollination). Transcripts for NAAT1 and AAT14 were present at highly distinct levels and accumulated during late developmental stages of cucumber plants. Transcript levels for NAAT1 were highest in the tissues collected at day 51 of the experiment and in fruits irrespective of the maturation. Transcripts encoding AAT14 were most abundant in stem collected at day 51 of the experiment and in fruit samples 8 days after pollination. Unlike the other three AAT genes, AAT9 showed very low expression in fruits and transcripts were observed in young tissues and in roots collected at day 51.

### Expression Patterns of Cucumber AAT Genes in Response to Abiotic Stresses

Plants at the two-true-leaf stage (approximately 2 weeks old) were subjected to cold, salt, drought stress, and ABA treatment. The transcript levels of four cucumber genes (AAT4, NAAT1, AAT9, and AAT14) were analyzed at different time points after treatment and compared to non-treated control plants (Supplementary Figure 5). The results showed that the expression of the AAT genes is regulated by different abiotic stresses although various expression profiles were observed for each gene against different abiotic stresses. Among the amaranthin-like genes studied, NAAT1, AAT9, and AAT14 are responsive to abiotic stresses/hormone treatment with NAAT1 being the most responsive. In contrast, AAT4 expression showed little or no changes after different stresses.

### Sequence Analysis and Molecular Modeling of AAT4

**FIGURE 8:**
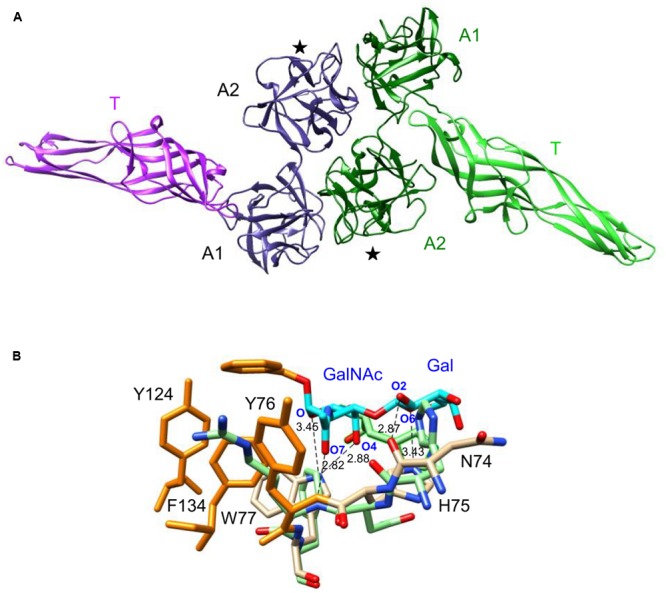
Molecular modeling of AAT4. **(A)** Ribbon diagram of the dimeric structure of AAT4. Both monomers, colored violet and green, respectively, consist of two amaranthin domains (A1, A2), linked to an aerolysin-like domain (T). Domains A2 contain a carbohydrate-binding site (★). **(B)** Network of H-bonds (dashed black lines) anchoring the benzoyl T-antigen disaccharide (cyan and orange stick) to the carbohydrate-binding site of the amaranthin lectin. A stacking interaction occurs between the benzoyl ring and aromatic residues Y76, Y124, and F134 (orange sticks) located in the vicinity of the active site. The H-bond distances are indicated in Ångströms. Amino acid residues colored pale green correspond to residues of the AAT4 A2 domain, homologous to the amino acid residues forming the carbohydrate-binding site of amaranthin.

The modeled AAT4 consists of two tandemly arrayed amaranthin domains, A1 and A2, linked to an aerolysin-like domain, T (**Figure 8A**). In order to restore functional carbohydrate-binding sites at the interface between two adjacent amaranthin domains, like in the amaranthin lectin from *A. caudatus* (Transue et al., 1997), two AAT4 monomers most probably associate head to tail, to build up a functional dimer (**Figure 8A**). However, the superimposition of the amino acid residues forming the carbohydrate-binding sites of amaranthin in complex with benzoyl T-antigen (Transue et al., 1997) to the corresponding amino acid residues of the potential carbohydrate-binding sites of the AAT4 amaranthin domains, shows that all of these residues are different and some of them create a steric hindrance with the sugar (**Figure 8B**).

### Phylogenetic Relationships of Aerolysin Domain in AAT4 with Aerolysin Domains from Other Proteins

**FIGURE 9:**
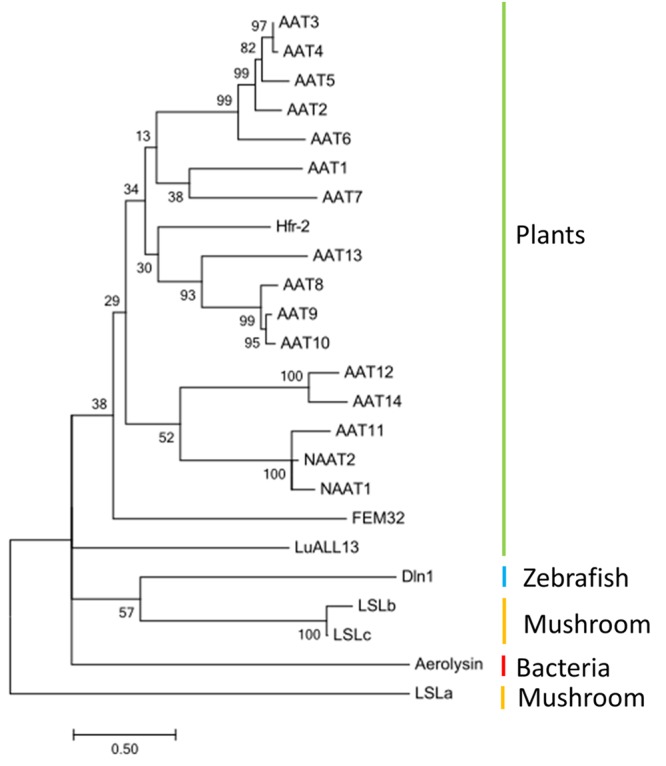
Phylogeny of aerolysin-like domain from different species: AAT1–AAT14, NAAT1, and NAAT2 (*Cucumis sativus*), Hfr-2 (*Triticum aestivum*), LuALL13 (*Linum usitatissimum*), FEM32 (*Rumex acetosa*), Aerolysin (*Aeromonas hydrophila*); Dln1 (*Danio rerio*), LSLa, LSLb, LSLc (*Laetiporus sulphureus*). The tree was built with MEGA6 and the bootstrap value next to the branches was estimated using the bootstrap test (500 replicates).

To understand the evolutionary relationships of the aerolysin domains from cucumber AATs with other known pore-forming proteins, a phylogenetic tree was built with sequences encoding the aerolysin-like domain from different species. A selection was made for sequences encoding several aerolysin domains that are known to be linked with lectin domains (**Figure 9**). The analysis showed that the AAT genes from cucumber are grouped together with plant sequences like Hfr-2 from wheat and FEM32 from *R. acetosa*. The LuAAL13 from flax is not close to other plant sequences and clustered together with the Dln1 sequence from zebrafish, the LSL sequence from mushroom and the aerolysin sequences from bacteria.

## Discussion

The analysis of different plant genomes revealed a large number of sequences with amaranthin domains. The number of amaranthin domains varies remarkably among different plant species. For some groups of plants (Rosales, Cucurbitaceae, Caryophyllales, Poaceae), amaranthin domains were identified in almost all sequenced genomes with only a few exceptions (**Figure 1**). In contrast, amaranthin-like sequences are absent in Fabaceae and Asterids (only one sequence retrieved from 10 plant genomes). Furthermore, based on our analysis for 84 sequenced plant genomes, it is obvious that the majority of the amaranthin-like sequences contain multiple protein domains with known function, the amaranthin domain being at least one of them. The aerolysin domain was found most frequently linked with amaranthin domains. It has been reported that genes containing aerolysin domains in eukaryotes may result from the recurrent horizontal transfer of bacterial toxin genes (Moran et al., 2012). Interestingly, some other protein domain combinations were found exclusively in one plant genome. For example, the Bet v1 domain and the TRAF-like domain were only found in flax and the fascin domain was only identified in *S. moellendorffii*. At present it cannot be excluded that these domain architectures might exist in plant genomes not included in this study, but they probably occur only in very few plant species.

Cucumber accommodates the largest number of AAT genes and therefore is a good choice for the study of the amaranthin-like proteins. Transcriptional analysis of amaranthin-like genes during the development of cucumber plants revealed specific expression profiles for AAT4, NAAT1, AAT9, and AAT14 in different tissues during plant development. For example, NAAT1 is expressed more in the older tissues while AAT9 is expressed in younger tissues. The tissue-specific expression of amaranthin-like proteins (with AAT domains) has been reported in two other plant species, flax and *R. acetosa*. LuALL13 from flax (gene corresponds to Lus10020808.g in our analysis) was found to be particularly enriched in floral tissues (Faruque et al., 2015) and the amaranthin-like protein FEM32 from *R. acetosa* was reported to be expressed specifically in flowers both at the early and late stages of development (Manzano et al., 2017). Despite the specific expression profiles for each particular AAT gene under study, transcripts for the amaranthin-like sequences were detected in all tissues throughout plant development, including cotyledons, leaves, stems, roots, flowers, and fruits.

In nature, cucumbers are very sensitive to low temperature (Chen et al., 2013), drought stress (Janoudi et al., 1993) and salinity stress (Zhong et al., 2016), which represent major threats that cucumbers have to cope with. The phytohormone ABA plays a critical role in the response to different stress conditions. ABA mainly functions as an endogenous messenger in the regulation of the water balance and osmotic stress tolerance. The application of ABA mimics the effect of a stress condition. Because many abiotic stresses ultimately result in desiccation of the cell and osmotic imbalance, there is an overlap in the expression of stress-related genes after cold, drought, salt, and ABA treatment (Tuteja, 2007). Stress experiments with cucumber seedlings showed that transcript levels for the AAT genes were changing in response to abiotic stresses such as cold, salt, drought, and the plant hormone ABA, suggesting that these proteins are involved in stress signaling. Different cucumber AAT genes exhibited different expression patterns for the abiotic stress/hormone treatments. These data suggest that these AAT proteins may play highly specific roles in order to help plants to cope with different unfavorable circumstances.

Recent studies showed that proteins with AAT domain architectures play different biological roles in plants. The Hfr-2 gene from wheat encodes a protein with AAT domain architecture (corresponds to Traes_2BS_9420D88C2.1 in this study) and its expression is up-regulated following infestation of virulent Hessian fly (*Mayetiola destructor*), fall armyworm (*Spodoptera frugiperda*), and bird cherry-oat aphid (*Rhopalosiphum padi*), but little or no changes in expression levels were observed after wounding, virus infection or chemical treatments with salicylic acid and ABA (Puthoff et al., 2005). The flower-specific FEM32, an AAT-like gene from *R. acetosa*, was reported to be involved in the sex determination and flower development (Manzano et al., 2017). Transgenic plants such as cotton and potato overexpressing amaranthin showed enhanced resistance against aphids, suggesting that amaranthin is involved in plant defense (Wu et al., 2006; Yang et al., 2011).

The modeling of AAT4 suggested that two AAT4 monomers might associate head to tail, to build up a functional dimer to restore the carbohydrate-binding sites of AAT4, similar to the amaranthin lectin (**Figure 8**). However, since the amino acids in the putative carbohydrate-binding sites of AAT4 are changed compared to the binding site in the *A. caudatus* lectin it is impossible to draw conclusions. Obviously, other types of oligomerization between AAT monomers may also be hypothesized based on protein association similar to the aerolysin from *Aeromonas* sp. (Degiacomi et al., 2013), the aerolysin-containing lectin from *L. sulphureus* (Tateno and Goldstein, 2003; Mancheno et al., 2005), or Dln1 from *D. rerio* (Jia et al., 2016).

Lectin domains linked to an aerolysin domain have been reported to be functional in several proteins. The *L. sulphureus* lectin purified from mushrooms consists of a β-trefoil scaffold resembling the ricin B domain, which specifically recognizes lactose, *N*-acetyl-D-lactosamine and other galactose-related saccharides (Mancheno et al., 2005). Another hemolytic lectin Dln1 contains a jacalin-related domain, which binds to high-mannose glycans (Jia et al., 2016). These proteins are structurally similar to AAT4 and were also used for the molecular modeling of AAT4. Although several AAT-like proteins (Hfr-2, FEM32) have been reported, none of these proteins has been characterized with respect to its biological activities, e.g., lectin activity and pore-forming activity. In future experiments, the biological activities of amaranthin-like proteins need to be investigated in detail aiming at a biochemical study of the biological activities of the lectin as well as the aerolysin domains. Currently, the lectin activity of amaranthin domains has only been investigated for lectins purified from the *Amaranthus* species (*A. caudatus, Amaranthus leucocarpus, Amaranthus hypochondriacus*). These amaranthin domains specifically interact with T-antigen and *N*-acetylgalactosamine (Ozeki et al., 1996; Transue et al., 1997; Hernández et al., 2001). Considering the significant sequence differences between all identified amaranthin domain sequences, it is likely that the carbohydrate-binding specificities for some of these amaranthin domains might be altered or could even be lost as a result of sequence variation and altered protein folding. Changes in the carbohydrate-binding specificity of the amaranthin domains could also allow the interaction with different glycans, possibly resulting in other interactions or more specific biological roles. Furthermore, taking into consideration the presence of multiple domain architectures in the amaranthin-like proteins it is likely that all these amaranthin homologs exert different biological activities and physiological roles.

The pore-forming process of aerolysin-like proteins is usually triggered in response to certain factors, such as proteolysis or pH change. Consequently, the proteins get activated, and multiple monomers of these proteins oligomerize into a pore structure (heptamer for aerolysin, hexamer for LSL, octamer for Dln1) upon binding of certain receptors on the cell membrane. After a conformational change, part of the aerolysin domain will be inserted into the cell membrane, resulting in pore formation in the target cell membrane and facilitating bacterial invasion (Iacovache et al., 2010; Degiacomi et al., 2013; Jia et al., 2016).

To our knowledge, this is the first study that describes the overall distribution of amaranthin-like genes and the diversity of their domain architectures. The investigation of amaranthin-like proteins from cucumber further expanded our knowledge on their subcellular localization, tissue-specific expression and their possible biological function. Considering the lack of research on this family of lectins, this work provided valuable information for future studies on amaranthin-like proteins and their physiological roles in plants.

## Author Contributions

LD and EVD outlined and designed the study. LD performed the experiments, analyzed and interpreted the data, and prepared the first draft of the manuscript. PR conducted the molecular modeling. EVD conceived and supervised the experiments and critically revised the manuscript. All authors have read, revised, and approved the final manuscript.

## Conflict of Interest Statement

The authors declare that the research was conducted in the absence of any commercial or financial relationships that could be construed as a potential conflict of interest.

## Footnotes

1 http://phylot.biobyte.de/

2 http://itol.embl.de/

3 https://www.ncbi.nlm.nih.gov/Taxonomy/CommonTree/wwwcmt.cgi

4 http://fiji.sc/

5 http://primer3.ut.ee/

6 http://www.swissdock.ch

## Abbreviations

ABA: abscisic acid
EGFP: enhanced green fluorescent protein

## References

[B1] Akiba T. Abe Y. Kitada S. Kusaka Y. Ito A. Ichimatsu T. (2009). Crystal structure of the parasporin-2 *Bacillus thuringiensis* toxin that recognizes cancer cells. *J. Mol. Biol.* 386 121–133. 10.1016/j.jmb.2008.12.002 19094993

[B2] Andre S. Kaltner H. Manning J. C. Murphy P. V. Gabius H. J. (2015). Lectins: getting familiar with translators of the sugar code. *Molecules* 20 1788–1823. 10.3390/molecules20021788 25621423 PMC6272290

[B3] Capella-Gutierrez S. Silla-Martinez J. M. Gabaldon T. (2009). trimAl: a tool for automated alignment trimming in large-scale phylogenetic analyses. *Bioinformatics* 25 1972–1973. 10.1093/bioinformatics/btp348 19505945 PMC2712344

[B4] Chen S. C. Jin W. J. Liu A. R. Zhang S. J. Liu D. L. Wang F. H. (2013). Arbuscular mycorrhizal fungi (AMF) increase growth and secondary metabolism in cucumber subjected to low temperature stress. *Sci. Hortic.* 160 222–229. 10.1016/j.scienta.2013.05.039

[B5] Dang L. Van Damme E. J. M. (2016). Genome-wide identification and domain organization of lectin domains in cucumber. *Plant Physiol. Biochem.* 108 165–176. 10.1016/j.plaphy.2016.07.009 27434144

[B6] Degiacomi M. T. Lacovache I. Pernot L. Chami M. Kudryashev M. Stahlberg H. (2013). Molecular assembly of the aerolysin pore reveals a swirling membrane-insertion mechanism. *Nat. Chem. Biol.* 9 623–629. 10.1038/Nchembio.1312 23912165

[B7] Edgar R. C. (2004). MUSCLE: multiple sequence alignment with high accuracy and high throughput. *Nucleic Acids Res.* 32 1792–1797. 10.1093/nar/gkh340 15034147 PMC390337

[B8] Faruque K. Begam R. Deyholos M. K. (2015). The amaranthin-like lectin (LuALL) genes of flax: a unique gene family with members inducible by defence hormones. *Plant Mol. Biol. Report.* 33 731–741. 10.1007/s11105-014-0791-4

[B9] Geny B. Popoff M. R. (2006). Bacterial protein toxins and lipids: pore formation or toxin entry into cells. *Biol. Cell* 98 667–678. 10.1042/Bc20050082 17042742

[B10] Grosdidier A. Zoete V. Michielin O. (2011a). Fast docking using the CHARMM force field with EADock DSS. *J. Comput. Chem.* 32 2149–2159. 10.1002/jcc.21797 21541955

[B11] Grosdidier A. Zoete V. Michielin O. (2011b). SwissDock, a protein-small molecule docking web service based on EADock DSS. *Nucleic Acids Res.* 39 W270–W277. 10.1093/nar/gkr366 21624888 PMC3125772

[B12] Hall B. G. (2013). Building phylogenetic trees from molecular data with MEGA. *Mol. Biol. Evol.* 30 1229–1235. 10.1093/molbev/mst012 23486614

[B13] Hellemans J. Mortier G. De Paepe A. Speleman F. Vandesompele J. (2007). qBase relative quantification framework and software for management and automated analysis of real-time quantitative PCR data. *Genome Biol.* 8:R19 10.1186/Gb-2007-8-2-R19 PMC1852402 17291332

[B14] Hernández P. Debray H. Jaekel H. Garfias Y. Carmen Jiménez Mdel C. Martínez-Cairo S. (2001). Chemical characterization of the lectin from *Amaranthus leucocarpus syn. hypocondriacus* by 2-D proteome analysis. *Glycoconj. J.* 18 321–329. 10.1023/A:1013760915738 11788800

[B15] Iacovache I. Bischofberger M. van der Goot F. G. (2010). Structure and assembly of pore-forming proteins. *Curr. Opin. Struct. Biol.* 20 241–246. 10.1016/j.sbi.2010.01.013 20172710

[B16] Janoudi A. K. Widders I. E. Flore J. A. (1993). Water deficits and environmental factors affect photosynthesis in leaves of cucumber (*Cucumis sativus*). *J. Am. Soc. Hortic. Sci.* 118 366–370.

[B17] Jia N. Liu N. Cheng W. Jiang Y. L. Sun H. Chen L. L. (2016). Structural basis for receptor recognition and pore formation of a zebrafish aerolysin-like protein. *EMBO Rep.* 17 235–248. 10.15252/embr.201540851 26711430 PMC5290818

[B18] Karimi M. Inze D. Depicker A. (2002). GATEWAY vectors for *Agrobacterium*-mediated plant transformation. *Trends Plant Sci.* 7 193–195. 10.1016/S1360-1385(02)02251-3 11992820

[B19] Krieger E. Koraimann G. Vriend G. (2002). Increasing the precision of comparative models with YASARA NOVA-a self-parameterizing force field. *Proteins* 47 393–402. 10.1002/prot.10104 11948792

[B20] Krogh A. Larsson B. von Heijne G. Sonnhammer E. L. L. (2001). Predicting transmembrane protein topology with a hidden Markov model: application to complete genomes. *J. Mol. Biol.* 305 567–580. 10.1006/jmbi.2000.4315 11152613

[B21] Lannoo N. Van Damme E. J. M. (2014). Lectin domains at the frontiers of plant defense. *Front. Plant Sci.* 5:397 10.3389/fpls.2014.00397 PMC4131498 25165467

[B22] Letunic I. Bork P. (2016). Interactive tree of life (iTOL) v3: an online tool for the display and annotation of phylogenetic and other trees. *Nucleic Acids Res.* 44 W242–W245. 10.1093/nar/gkw290 27095192 PMC4987883

[B23] Lichtenstein R. G. Rabinovich G. A. (2013). Glycobiology of cell death: when glycans and lectins govern cell fate. *Cell Death Differ.* 20 976–986. 10.1038/cdd.2013.50 23703323 PMC3705604

[B24] Mancheno J. M. Tateno H. Goldstein I. J. Martinez-Ripoll M. Hermoso J. A. (2005). Structural analysis of the *Laetiporus sulphureus* hemolytic pore-forming lectin in complex with sugars. *J. Biol. Chem.* 280 17251–17259. 10.1074/jbc.M413933200 15687495

[B25] Manzano S. Megias Z. Martinez C. Garcia A. Aguado E. Chileh T. (2017). Overexpression of a flower-specific aerolysin-like protein from the dioecious plant *Rumex acetosa* alters flower development and induces male sterility in transgenic tobacco. *Plant J.* 89 58–72. 10.1111/tpj.13322 27599169

[B26] Migocka M. Papierniak A. (2011). Identification of suitable reference genes for studying gene expression in cucumber plants subjected to abiotic stress and growth regulators. *Mol. Breed.* 28 343–357. 10.1007/s11032-010-9487-0

[B27] Moran Y. Fredman D. Szczesny P. Grynberg M. Technau U. (2012). Recurrent horizontal transfer of bacterial toxin genes to eukaryotes. *Mol. Biol. Evol.* 29 2223–2230. 10.1093/molbev/mss089 22411854 PMC3424411

[B28] Mulder N. Apweiler R. (2007). InterPro and InterProScan: tools for protein sequence classification and comparison. *Methods Mol. Biol.* 396 59–70. 10.1007/978-1-59745-515-2_5 18025686

[B29] Ozeki M. Kamemura K. Moriyama K. Itoh Y. Furuichi Y. Umekawa H. (1996). Purification and characterization of a lectin from *Amaranthus hypochondriacus* var. Mexico Seeds. *Biosci. Biotechnol. Biochem.* 60 2048–2051. 10.1271/bbb.60.2048 8988637

[B30] Parker M. W. Buckley J. T. Postma J. P. M. Tucker A. D. Leonard K. Pattus F. (1994). Structure of the *Aeromonas* toxin proaerolysin in its water-soluble and membrane-channel states. *Nature* 367 292–295. 10.1038/367292a0 7510043

[B31] Petersen T. N. Brunak S. von Heijne G. Nielsen H. (2011). SignalP 4.0: discriminating signal peptides from transmembrane regions. *Nat. Methods* 8 785–786. 10.1038/nmeth.1701 21959131

[B32] Pettersen E. F. Goddard T. D. Huang C. C. Couch G. S. Greenblatt D. M. Meng E. C. (2004). UCSF Chimera- A visualization system for exploratory research and analysis. *J. Comput. Chem.* 25 1605–1612. 10.1002/jcc.20084 15264254

[B33] Peumans W. J. Van Damme E. J. M. (1995). Lectins as plant defense proteins. *Plant Physiol.* 109 347–352. 10.1104/Pp.109.2.347 7480335 PMC157596

[B34] Pfaffl M. W. Horgan G. W. Dempfle L. (2002). Relative expression software tool (REST) for group-wise comparison and statistical analysis of relative expression results in real-time PCR. *Nucleic Acids Res.* 30 e36. 10.1093/nar/30.9.e36 PMC113859 11972351

[B35] Puthoff D. P. Sardesai N. Subramanyam S. Nemacheck J. A. Williams C. E. (2005). Hfr-2, a wheat cytolytic toxin-like gene, is up-regulated by virulent Hessian fly larval feeding. *Mol. Plant Pathol.* 6 411–423. 10.1111/J.1364-3703.2005.00289.X 20565667

[B36] Stamatakis A. Hoover P. Rougemont J. (2008). A rapid bootstrap algorithm for the RAxML Web servers. *Syst. Biol.* 57 758–771. 10.1080/10635150802429642 18853362

[B37] Stolzer M. Lai H. Xu M. Sathaye D. Vernot B. Durand D. (2012). Inferring duplications, losses, transfers, and incomplete lineage sorting with non-binary species trees. *Bioinformatics* 28 i409–i415. 10.1093/bioinformatics/bts386 22962460 PMC3436813

[B38] Szczesny P. Iacovache I. Muszewska A. Ginalski K. van der Goot F. G. Grynberg M. (2011). Extending the aerolysin family: from bacteria to vertebrates. *PLoS ONE* 6:e20349 10.1371/journal.pone.0020349 PMC3110756 21687664

[B39] Tateno H. Goldstein I. J. (2003). Molecular cloning, expression, and characterization of novel hemolytic lectins from the mushroom *Laetiporus sulphureus*, which show homology to bacterial toxins. *J. Biol. Chem.* 278 40455–40463. 10.1074/jbc.M306836200 12900403

[B40] Transue T. R. Smith A. K. Mo H. Goldstein I. J. Saper M. A. (1997). Structure of benzyl T-antigen disaccharide bound to *Amaranthus caudatus* agglutinin. *Nat. Struct. Biol.* 4 779–783. 10.1038/nsb1097-779 9334739

[B41] Tuteja N. (2007). Abscisic acid and abiotic stress signaling. *Plant Signal. Behav.* 2 135–138. 10.4161/psb.2.3.4156 19516981 PMC2634038

[B42] Van Damme E. J. M. (2014). History of plant lectin research. *Methods Mol. Biol.* 1200 3–13. 10.1007/978-1-4939-1292-6_1 25117220

[B43] Van Damme E. J. M. Lannoo N. Peumans W. J. (2008). Plant lectins. *Adv. Bot. Res.* 48 107–209. 10.1016/s0065-2296(08)00403-5

[B44] Wu J. Luo X. Guo H. Xiao J. Tian Y. (2006). Transgenic cotton, expressing *Amaranthus caudatus* agglutinin, confers enhanced resistance to aphids. *Plant Breed.* 125 390–394. 10.1111/j.1439-0523.2006.01247.x

[B45] Yang X. Zhang X. R. Zhang M. J. Guo W. C. Tian Y. C. Zhong Q. (2011). Transgenic potato overexpressing the *Amaranthus caudatus* agglutinin gene to confer aphid resistance. *Crop Sci.* 51 2119–2124. 10.2135/cropsci2010.11.0650

[B46] Zhong M. Yuan Y. H. Shu S. Sun J. Guo S. R. Yuan R. N. (2016). Effects of exogenous putrescine on glycolysis and Krebs cycle metabolism in cucumber leaves subjected to salt stress. *Plant Growth Regul.* 79 319–330. 10.1007/s10725-015-0136-9

