# Supplementary material for: Amaranthin-Like Proteins with Aerolysin Domains in Plants
Source: Front Plant Sci. 2017 Aug 10;8:1368. doi: 10.3389/fpls.2017.01368 (PMC5554364; doi:10.3389/fpls.2017.01368)
Supplement: Supplementary file 1 [file Data_Sheet_1.PDF]

Supplementary Material

Distribution of amaranthin-like proteins and their expression analysis in cucumber

Liuyi Dang[1], Pierre Rougé[2], Els J.M. Van Damme[1*]

*** Correspondence:** Prof. dr. Els Van Damme: Elsjm.VanDamme@UGent.be

1 Supplementary file 1

Plant genome databases used to identify amaranthin domains:

Phytozome v 11 http://www.phytozome.net

NCBI genome http://www.ncbi.nlm.nih.gov/genome

Sol Genomics Network https://solgenomics.net

Genome Database for Rosaceae https://www.rosaceae.org/

Ensemble plants http://plants.ensembl.org

LOTUS-DB http://lotus-db.wbgcas.cn

Genlisea genome database http://genlisea.org/

Genome database for *Vaccinium* https://www.vaccinium.org

Coffee Genome Hub http://coffee-genome.org

Kiwifruit Genome Database http://bioinfo.bti.cornell.edu/cgi-bin/kiwi/home.cgi

The *Beta vulgaris* resource http://bvseq.molgen.mpg.de/index.shtml

Jatropha Genome Database http://www.kazusa.or.jp/jatropha/

Melonomics https://melonomics.net/

Congenie.org http://congenie.org/

Bamboo Genome Database http://www.bamboogdb.org/

The Dwarf Birch Genome Project http://birchgenome.org

Thellungiella http://thellungiella.org/

Chickpea Genomic Web Resource http://www.nipgr.res.in/CGWR

Miyakogusa.jp http://www.kazusa.or.jp/lotus/index.html

Peanut Base http://peanutbase.org

Brassica database http://brassicadb.org

Pico PLAZA 2.0 http://bioinformatics.psb.ugent.be/plaza/versions/pico-plaza/

Klebsormidium flaccidum genome project
http://www.plantmorphogenesis.bio.titech.ac.jp/~algae_genome_project/klebsormidium/

2 Supplementary Figures and Tables

2.1 Supplementary Figures

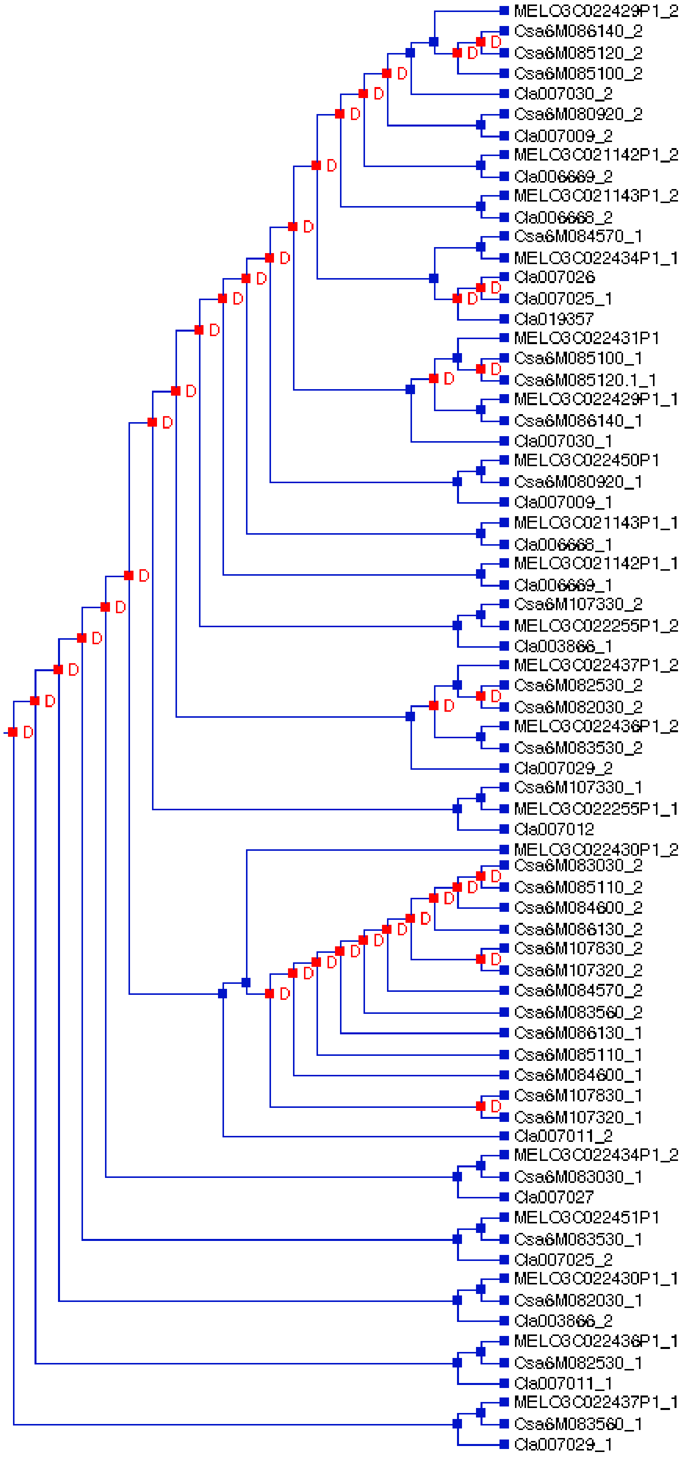

Image generated with Notung 2.9, on Jul 15, 2017

Supplementary Figure 1. Reconciliated tree. Phylogenetic tree of the amaranthin domains reconciliated with the species tree using Notung 2.9 (http://www.cs.cmu.edu/~durand/Notung/). Sequences with two amaranthin domains were separated into two sequences. Numbers after the sequence name refer to amaranthin domain 1 or 2. Duplication events are indicated with “D”.

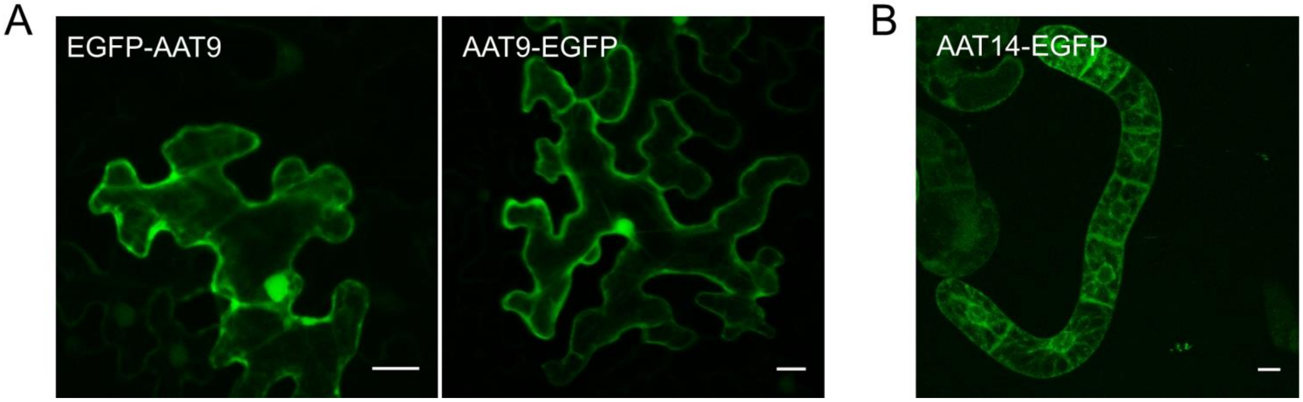

Supplementary Figure 2. Confocal images of EGFP fusion proteins for different AAT sequences from cucumber. (A) transiently transformed tobacco leaves with expression of EGFP-AAT9 and AAT9-EGFP; (B) stably transformed tobacco suspension BY-2 cells with expression of AAT14-EGFP.

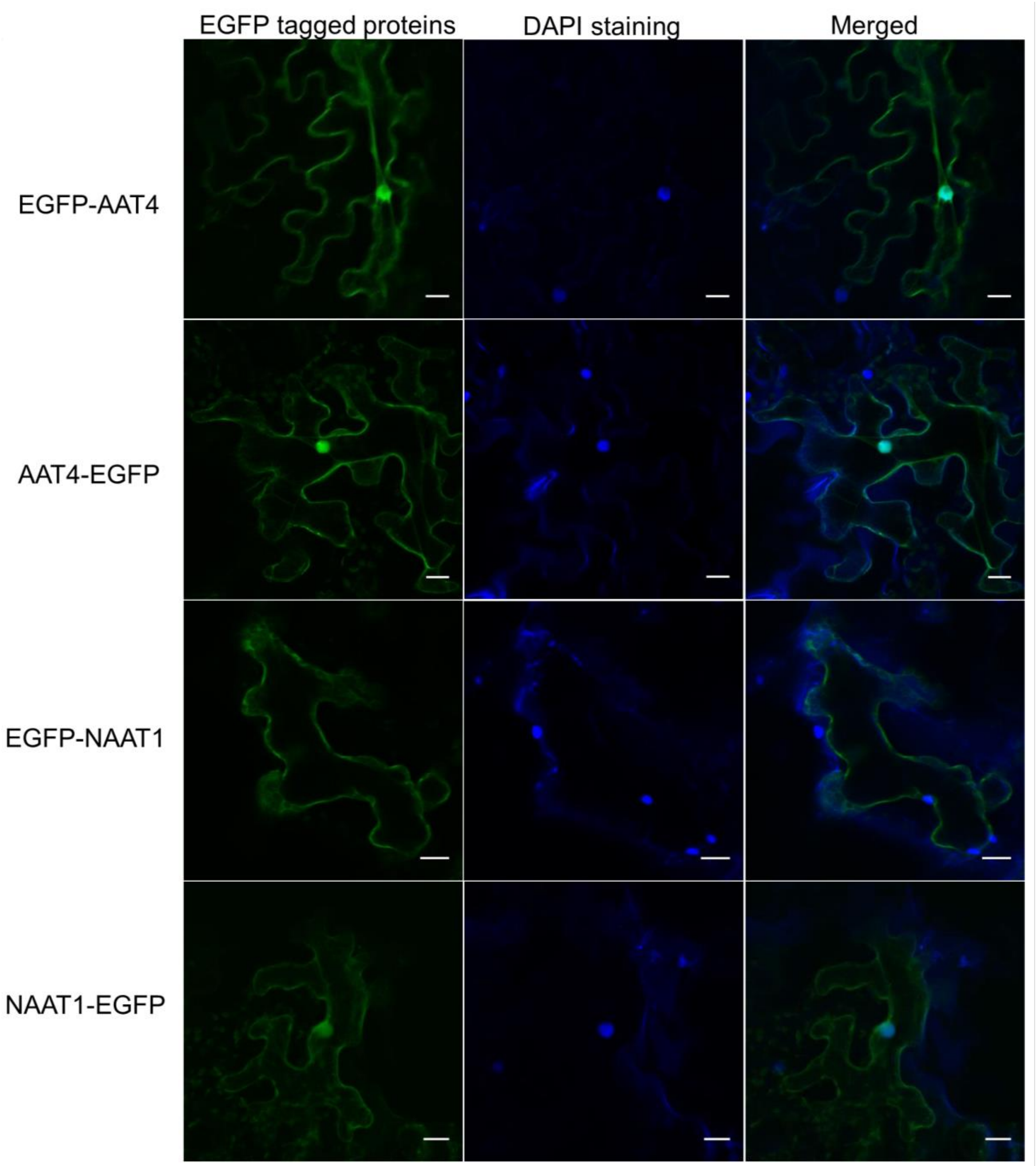

Supplementary Figure 3. Confocal images of AAT4 and NAAT1 fused with EGFP transiently expressed in tobacco leaves with DAPI counterstaining. Scale bars represent 20 nm.

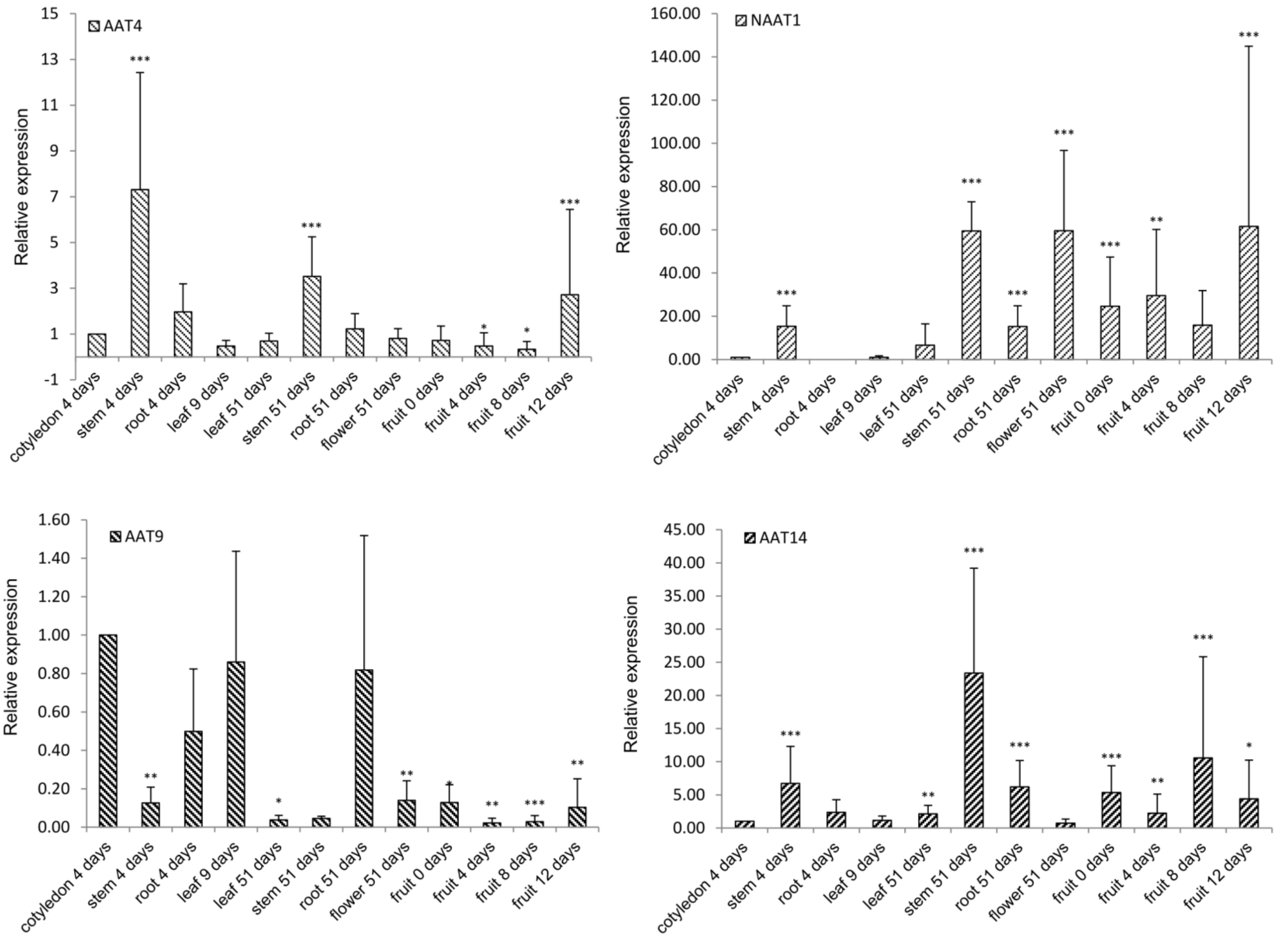

Supplementary Figure 4. Transcriptional profiling of AAT genes in different tissues during cucumber development. Relative expression levels of AAT genes are compared to the expression level in cotyledons collected at day 4 of cucumber development. Expression levels compared between four AAT genes in cotyledon: AAT4 : NAAT1 : AAT9 : AAT14 are approximately 1 : 0.01 : 1.49 : 0.04. Bars represent means and standard errors from two biological replicates, each replicate containing a pool of 3 plants. Asterisks indicated statistically significant differences compared with the control tissue (* $p \leqslant 0.05$, ** $p \leqslant 0.01$, ***$p \leqslant 0.001$; REST analysis).

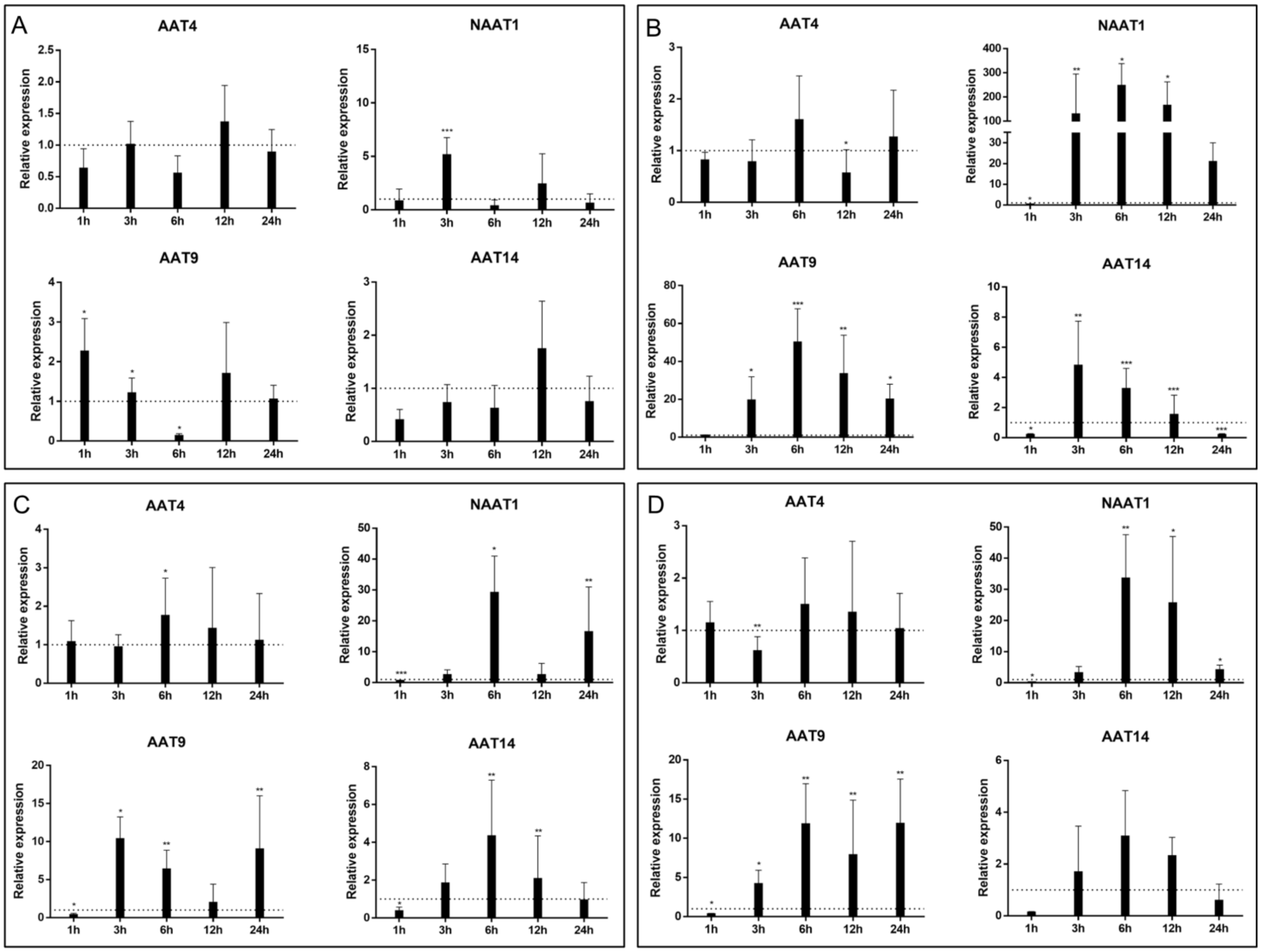

Supplementary Figure 5. Relative transcript levels for AAT genes in cucumber plants subjected to different abiotic stress conditions. (A) Cold treatment; (B) Salt treatment; (C) Drought treatment; (D) ABA treatment. Bars represent means and standard errors from two independent biological replicates, each replicate containing a pool of 4 plants. Asterisks indicate statistically significant differences compared with the control treatment (* p≤0.05, ** p≤0.01, ***p≤0.001; REST analysis).

2.2 Supplementary Tables

Supplementary Table 1. Primers for amplification of sequences encoding cucumber AAT genes, to make EGFP-fusion constructs

Gene Name	Forward primer (5'-3')	Reverse primer (5'-3')
**AAT4**	AAAAAGCAGGCTTCACCATGTCGT TTCTTCCTAAGTTTG	AGAAAGCTGGGTGGCGGTGT GATGGTGAAT
**NAAT1**	AAAAAGCAGGCTTCACCATGAATC TTTTCAAAGGATTGG	AGAAAGCTGGGTGCAATGATT CTACTTTTTCAGTC
**AAT9**	AAAAAGCAGGCTTCACCATGGAG GAAGAAAGCCAATTG	A GAA AGC TGG GTGGAG ATC TTC TTG CTC TGTGG
**AAT14**	AAAAAGCAGGCTTCACCATGATTC CAA GTTTACCTCCTC	AGAAAGCTGGGTCTATAGGTA GTGCTTCCTCATC
**Primers to complete attB site**	GGGGACAAGTTTGTACAAAAAAG CAGGCT	GGGGACCACTTTGTACAAGA AAGCTGGGT

Supplementary Table 2. Primers for qPCR analysis

**Gene**	**Forward primer (5'-3')**	**Reverse primer (5'-3')**
**CACS**	TGGGAAGATTCTTATGAAGTGC	CTCGTCAAATTTACACATTGGT
**PP2A**	CAACAGGTGATATTGGATTATGATTATAC	GCCAGCTCATCCTCATATAAG
**AAT4**	TTCGTACACGCAACGAGA	TGAAGAGGGTAAGGCTTG
**NAAT1**	GAGGAGCTGTGAAAGGAGCA	CCCTCCACGACAGTTCCAAT
**AAT9**	CAGAAACAGCGAACCAGAGC	AACTTCATCCCCACCGAGTT
**AAT14**	GGGAATAGAGACGATCCGAACT	GCGCAGAAGGCAGTGTTT

Supplementary Table 3. List of amaranthin-like genes in 34 plant genomes

**Plant species (Common name)**	**Accession No. in the genome database**	**Domain architecture**	**Length (aa)**
Actinidia chinensis (Kiwifruit)	Achn294821	AAT	465
Aegilops tauschii (Tausch's goatgrass)	EMT28372	AAT	493
	EMT27991	nAAT	602
	EMT22646	nAT	378
	EMT22647	A	128
	EMT26723	AT	317
	EMT26724	A	145
Amaranthus hypochondriacus (Prince's feather)	AHYPO_005380	AA	267
	AHYPO_007409	AA	263
	AHYPO_007410	A	92
	AHYPO_009184	nA	253
	AHYPO_014015	AATc	526
	AHYPO_014017	AATc	518
	AHYPO_014800	AA	285
	AHYPO_018341	AA	300
	AHYPO_018344	AAc	308
Aquilegia coerulea (Columbine)	Aqcoe4G310000	AAT	445
	Aqcoe7G375600	AAT	474
Beta vulgaris (Sugar beet)	Bv_012850_dfio.t1	AAT	476
	Bv1_005860_xikd.t1	AAT	439
	Bv2_039060_whdc.t1	AATc	533
	Bv2_039110_mrmn.t1	AATc	533
	Bv4_082910_yeau.t1	AA	302
	Bv6_147110_njcr.t1	AAT	497
	Bv6_147160_zxyi.t1	AAT	496
	Bv8_188280_zqur.t1	AAT	506
	Bv9_213380_kxhh.t1	AA	299
	Bv9_224070_ycao.t1	AA	303
Brachypodium distachyon (Purple false brome)	Bradi1g02205.1	AAT	426
	Bradi1g02205.2	AA	349
	Bradi1g56260.1	AAT	517
	Bradi1g56260.2	nAT	404
	Bradi2g15370	AAT	542
	Bradi4g13580	AAT	487
Brachypodium stacei	Brast06G204800	AA	230
	Brast06G205000	AAT	444

Carica papaya (Papaya)	evm.TU.contig_29394.3	A	38
	evm.TU.contig_30822.2	AA	202
	evm.TU.contig_34789	AAT	306
	evm.TU.contig_44888	AA	258
	evm.TU.supercontig_125.1	AT	233
	evm.TU.supercontig_125.2	A	168
	evm.TU.supercontig_1831.1	AAT	465
	evm.TU.supercontig_223.19	AAT	421
	evm.TU.supercontig_264.7	AAT	465
	evm.TU.supercontig_264.8	AAT	465
	evm.TU.supercontig_291.2	AAT	471
	evm.TU.supercontig_291.3	A	96
	evm.TU.supercontig_567.1	AAT	468
	evm.TU.supercontig_567.2	A	122
	evm.TU.supercontig_83.14	AAT	468
	evm.TU.supercontig_83.16	AT	178
	evm.TU.supercontig_83.17	AAT	467
	evm.TU.supercontig_83.19	A	197
Citrullus lanatus (Watermelon)	Cla003866	AAT	475
	Cla006668	AAT	462
	Cla006669	AAT	466
	Cla007009	AAT	479
	Cla007011	AAT	448
	Cla007012	AT	337
	Cla007025	AAT	472
	Cla007026	A	155
	Cla007027	ATc	289
	Cla007029	AAT	474
	Cla007030	nAAT	629
	Cla019357	A	187
Cucumis melo (Melon)	MELO3C021142P1	AAT	468
	MELO3C021143P1	AAT	460
	MELO3C022255P1	AAT	473
	MELO3C022429P1	nAAT	610
	MELO3C022430P1	AAT	474
	MELO3C022431P1	nA	322
	MELO3C022434P1	AAT	477
	MELO3C022436P1	AAT	466
	MELO3C022437P1	AAT	463
	MELO3C022450P1	A	189
	MELO3C022451P1	A	186
Cucumis sativus (Cucumber)	Csa6M080920	AAT	484
	Csa6M082030	AAT	463

	Csa6M082530	AAT	463
	Csa6M083030	AAT	466
	Csa6M083530	AAT	463
	Csa6M083560	AAT	464
	Csa6M084570	AAT	471
	Csa6M084600	AAT	475
	Csa6M085100	nAAT	626
	Csa6M085110	AAT	474
	Csa6M085120	nAAT	614
	Csa6M086130	AAT	471
	Csa6M086140	nAAT	476
	Csa6M107320	nAAT	502
	Csa6M107330	AAT	468
	Csa6M107830	nAAT	505
Fragaria vesca (Woodland strawberry)	mrna00459.1-v1.0-hybrid	AAT	492
	mrna03811.1-v1.0-hybrid	HAATc	778
	mrna04189.1-v1.0-hybrid	AATc	530
	mrna04191.1-v1.0-hybrid	AATc	532
	mrna09165.1-v1.0-hybrid	AAT	504
	mrna09225.1-v1.0-hybrid	AAT	593
	mrna09339.1-v1.0-hybrid	AAT	498
	mrna22562.1-v1.0-hybrid	AAT	519
	mrna31830.1-v1.0-hybrid	AAT	511
	mrna33785.1-v1.0-hybrid	AA	323
	mrna33786.1-v1.0-hybrid	AATc	545
Gossypium raimonddi (Cotton)	Gorai.005G011400	AAAT	637
	Gorai.005G011500	AAT	474
	Gorai.005G011800.1	AAAT	618
	Gorai.005G011800.2	nAAT	495
	Gorai.005G011900	AAAT	602
	Gorai.005G012000	AAT	501
	Gorai.005G012100	AAT	473
	Gorai.005G023100	AAT	482
	Gorai.005G024800	AAT	455
Hordeum vulgare (Barley)	F2CYR6	AAT	540
	M0UVJ6	A	132
	M0UVJ7	AAT	508
	M0UVJ8	AAT	509
	M0UVK0	AT	363
	M0V4P6	AA	384
	M0ZD60	AAT	479
Jatropha curcas	Jcr4S02461.40	AAT	472

(Barbados nut)	Jcr4S01319.40	AAT	450
	Jcr4S02168.30	AAT	462
	Jcr4S01319.50	AAT	465
	Jcr4S02168.40	AAT	459
	Jcr4S00367.30	AATc	521
	Jcr4S01561.20	A	193
	Jcr4S21381.10	AZ	271
Linum usitatissimum (Flax)	Lus10002735.g	AAT	559
	Lus10005395.g	Ac	226
	Lus10005397.g	Ac	219
	Lus10005398.g	A	194
	Lus10008233.g	AR	423
	Lus10010698.g	nAB	464
	Lus10010702.g	AB	717
	Lus10016107.g	A	177
	Lus10016109.g	AAT	503
	Lus10020249.g	AAT	483
	Lus10020808.g	nAAT	510
	Lus10021453.g	A	120
	Lus10022894.g	Ac	255
	Lus10024084.g	Ac	231
	Lus10024931.g	Ac	264
	Lus10024934.g	AAT	475
	Lus10025503.g	AAT	484
	Lus10026716.g	ATc	313
	Lus10029182.g	AB	410
	Lus10029184.g	AAB	580
	Lus10029186.g	AAB	1024
	Lus10030648.g	A	232
	Lus10031082.g	AAT	493
	Lus10041636.g	A	233
Malus domestica (Apple)	MDP0000134302	A	165
	MDP0000149649	A	163
	MDP0000166067	A	164
	MDP0000167664	A	165
	MDP0000186108	A	202
	MDP0000186287	A	165
	MDP0000192309	A	165
	MDP0000198856	A	165
	MDP0000210676	A	165
	MDP0000258125	A	159
	MDP0000277475	A	153
	MDP0000278101	Ac	232

	MDP0000295606	AA	322
	MDP0000380205	AAT	498
	MDP0000426531	Ac	252
	MDP0000568858	AATc	658
	MDP0000732429	AAA	486
	MDP0000764263	AAA	480
	MDP0000772451	AA	345
	MDP0000816071	AA	300
	MDP0000138196	A	109
	MDP0000903965	AA	322
Morus notabilis (Mulberry)	XP_010095389	AAT	480
	XP_010092326	A	112
Panicum hallii (Hall's panicgrass)	Pahal.H00381	AAT	489
	Pahal.H02137	AAT	481
	Pahal.H02134	AAT	481
Panicum virgatum (Switchgrass)	Pavir.J08451	AT	348
	Pavir.J03404	AA	324
	Pavir.Ia02128	AAT	413
	Pavir.Hb01446	Ac	194
	Pavir.J23124	A	152
	Pavir.Ha00118	AAT	413
	Pavir.Hb01411	nAT	359
	Pavir.Ha00681	AAT	480
	Pavir.Hb01396	AT	233
Phyllostachys edulis (Moso bamboo)	PH01003566G0160	AATc	627
Picea abies (Norway spruce)	MA_22375g0010	AAT	338
	MA_9893972g0010	AT	316
Prunus mume (Chinese plum)	XP_008218989	AAT	519
	XP_008229256	A	158
	XP_008229291	A	163
	XP_008229293	A	145
	XP_008229341	A	158
	XP_008245072	A	158
	XP_008245433	AAT	466
	XP_008245434	AAT	436
	XP_008245435	AT	429
	XP_008245436	AAT	470
	XP_008245569	A	143
Prunus persica (Peach)	Prupe.2G087400	AAT	473
	Prupe.2G097500	AAT	519
	Prupe.2G097700	AAT	470

	Prupe.2G097800	AT	443
	Prupe.3G153100	A	158
	Prupe.3G153300	A	159
	Prupe.3G153600	A	157
	Prupe.5G237200	A	158
Pyrus bretschneideri (Pear)	XP_009341137	A	152
	XP_009342746	A	153
	XP_009342749	A	142
	XP_009343274	AA	327
	XP_009356088	AA	332
	XP_009360034	AAA	481
	XP_009368994	AA	390
	XP_009368995	AAT	498
	XP_009373857	AAT	492
	XP_009373858	AAT	529
	XP_009373871	AAT	529
	XP_009376017	AAT	475
Ricinus communis (Castor bean)	29737.t000040	AAT	472
	30078.t000143	AAT	466
	30078.t000144	AAT	476
	30169.t000182	AAT	394
Selaginella moellendorffii (Handsome spikemoss)	269784	AT	441
	402373	nFATc	551
	403095	nAT	560
	409476	AAT	496
	410027	AT	375
	424555	FAT	477
	437708	nAT	426
	447143	AAT	488
Setaria italica (Foxtail millet)	Seita.7G274200	AAT	527
Sataria viridis (Green foxtail)	Sevir.7G285200	AAT	527
Spinacia oleracea (Spinach)	Sp_096930_munp.t2	AAT	500
	Sp_096930_munp.t1	AAT	499
	Sp_001340_gcdp.t	AAT	500
	Sp_119720_znuk.t1	AAT	496
	Sp_119730_adif.t1	AAT	463
	Sp_173920_hcxt.t1	AT	454
	Sp_120900_tsqc.t1	AAT	457
	Sp_120890_gxdo.t1	AAT	431
	Sp_201840_mrro.t1	A	125
	Sp_043030_tnxq.t1	Ac	184
	Sp_056200_rpmu.t2	AT	353
Triticum aestivum	Traes_2BS_9420D88C2.1	AAT	492

(Wheat)	Traes_2DL_006976EC3	A	158
	Traes_4BL_5417BB190	A	126
	Traes_4BL_BCC3FB7E7.1	AT	295
	Traes_4BL_BCC3FB7E7.2	AT	295
	Traes_4BS_AE5CB4A8C	AT	295
	Traes_4BS_C0AABAF7B.1	A	130
	Traes_4BS_C0AABAF7B.2	A	90
	Traes_5AL_0E7B150F0	AT	295
	Traes_5BL_B80DDB1B7.1	AT	295
Vitis vinifera (Grape)	GSVIVG01028606001	AAT	465
	GSVIVG01028617001	AAT	467
	GSVIVG01028618001	AAAT	594
Zea mays (Maize)	GRMZM2G050131	nAT	374
	GRMZM2G056500	nAT	372
	GRMZM2G114945	nAT	374
	GRMZM2G134264	nAT	372

Note: n: N-terminal domain (>50 aa); c: C-terminal domain (>50 aa); A: amaranthin domain; T: aerolysin domain; F: fascin domain; B: Bet v1 domain; R: TRAF like domain; H: alpha/beta hydrolase domain, Z: B-box type zinc finger domain.
